# Impact of Equation Choice on Models Assessing the Association Between Lipoprotein(a) and Estimated Glomerular Filtration Rate in Adult Patients Without Chronic Kidney Disease

**DOI:** 10.3390/diseases14080277

**Published:** 2026-07-31

**Authors:** Irena Gencheva-Angelova, Radka Nuneva-Doncheva

**Affiliations:** 1 Department of Clinical Laboratory, Immunology, and Allergology, Medical University—Pleven, 1 Sv. Kliment Ohridski Str., 5800 Pleven, Bulgaria; gencheva1677@gmail.com; 2 Department of Clinical Laboratory, UMHAT Medica—Ruse, 35 Riga St., 7000 Ruse, Bulgaria

**Keywords:** lipoprotein(a), eGFR equations, CKD-EPI 2021, assessment models, comparative analysis

## Abstract

**Background:** In our recent research, we established a strong and statistically significant association between the highest quartile of lipoprotein(a) [Lp(a)] and mildly reduced estimated glomerular filtration rate (eGFR). Comparisons with similar studies were hindered by varying Lp(a) analytical methods and different eGFR equations. We analyzed how replacing the diagnostic standard CKD-EPI 2021 equation with alternatives (CKD-EPI 2009, CKD-MDRD, CKD-EKFC) affects predictive models under comprehensive demographic and clinical confounder adjustments. **Methods:** We calculated eGFR for 310 adults using the four equations. Creatinine and Lp(a) were measured via IFCC-recommended methods. The cohort was divided into a main (eGFR 60–80 mL/min/1.73 m^2^) and a control group (eGFR > 80 mL/min/1.73 m^2^). Multivariable logistic regression and Area Under the Curve (AUC) metrics evaluated model performance. **Results:** Alternative equations systematically underestimated eGFR, artificially increasing the reduced filtration group. After full multivariable adjustment, only CKD-EPI 2021 preserved the independent association between the highest Lp(a) quartile and mildly decreased eGFR (OR = 2.65, *p* = 0.008), achieving an AUC of 0.732. Conversely, CKD-EPI 2009 lost significance; CKD-MDRD exhibited mathematical instability from control group depletion, and EKFC showed severe over-adjustment when demographic covariates were included. **Conclusions:** Older equations and the age-embedded EKFC equation may influence regression models, potentially obscuring the true biomarker associations. CKD-EPI 2021 demonstrated the highest precision, successfully isolating physiological aging and suggesting an independent association between high Lp(a) and mildly reduced eGFR, irrespective of metabolic and vascular confounders.

## 1. Introduction

Accurate estimation of the glomerular filtration rate (eGFR) is fundamental for the diagnosis and staging of renal dysfunction in clinical practice. Currently, the race-independent CKD-EPI 2021 equation is accepted as the diagnostic standard to improve evaluation accuracy [1,2]. In contrast, older formulas (CKD-MDRD and CKD-EPI 2009), as well as the more recent CKD-EKFC equation, exhibit a tendency to underestimate eGFR [3]. This discrepancy becomes critical within the “gray zone” of low-normal kidney function, defined by an eGFR range of 60 to 89 mL/min/1.73 m^2^ [4]. Misclassification of patients within this range distorts clinical assessment and hinders the identification of early pathological processes.

Within this borderline zone, research interest is increasingly focused on identifying specific biomarkers predictive of early renal injury. Serum lipoprotein(a) [Lp(a)] is a well-established genetic risk factor for cardiovascular disease [5], but some recent studies also indicate its direct involvement in renal pathology [6]. A complex, bidirectional relationship exists between Lp(a) and kidney function: while renal impairment is known to elevate serum Lp(a) levels due to the kidneys’ role in its catabolism [7,8], high concentrations of this lipid conversely induce glomerular injury via pro-inflammatory and pro-thrombotic pathways [9,10].

In our study, we demonstrated a strong and statistically significant association between the highest Lp(a) quartile and low-normal eGFR in patients without manifest chronic kidney disease (CKD). However, directly comparing these findings with similar studies presents a major challenge. In available studies, authors utilize different eGFR equations, which alters cohort distribution, and frequently fail to adjust the statistical analysis for key confounding factors such as age.

Therefore, the objective of the present study was to conduct a comparative analysis to determine how replacing the recommended CKD-EPI 2021 standard with alternative equations (CKD-EPI 2009, CKD-MDRD, and CKD-EKFC) influences the final outcomes of multivariable logistic regression models evaluating the association between high serum Lp(a) levels and mildly reduced eGFR.

## 2. Materials and Methods

### 2.1. Subjects

The participant selection technique involved consecutive sampling of individuals who visited the laboratory and met the inclusion criteria, until the target sample size of 310 volunteers was reached. Each participant provided written informed consent. The primary inclusion criterion was age over 40 years. This threshold was selected because the physiological, non-linear decline in renal function typically becomes clinically measurable after the fourth decade of life [11]. Beyond this biological threshold, structurally normal kidneys experience a progressive reduction in nephron mass and renal perfusion, averaging a decline of approximately 0.8 to 1.0 mL/min/1.73 m^2^ per year [11]. Additional inclusion criteria required an eGFR ≥ 60 mL/min/1.73 m^2^ and the absence of CKD. Individuals with acute inflammation, acute vascular event, oncological disease, liver disease, and hyper- or hypothyroid conditions were excluded from the study. Individuals with missing data for any of the laboratory parameters included in the statistical analysis were also excluded from the study.

### 2.2. Methods

Creatinine and Lp(a) were measured using the methods recommended by the International Federation of Clinical Chemistry and Laboratory Medicine (IFCC) on a Cobas Pure analyzer (Roche Diagnostics GmbH, Mannheim, Germany). Serum creatinine was measured using an enzymatic assay with a calibrator traceable to an international reference material determined by isotope dilution mass spectrometry (IDMS) [12,13,14]. Lp (a) concentration was measured using the Tina-quant Lipoprotein(a) Gen.2 latex-enhanced immunoturbidimetric assay (Roche Diagnostics). The assay is independent of apo(a) isoform size, and its calibrator is traceable to the international reference material endorsed by IFCC [15,16]. The eGFR was calculated using all four equations: CKD-EPI 2021 [13], CKD-EPI 2009 [17], CKD-MDRD [18], and CKD-EKFC [19].

### 2.3. Statistical Analysis

For greater precision, the values of the independent variable Lp(a) were categorized into four quartiles (Q1, Q2, Q3, and Q4), with Q1 considered as the reference. The dependent variable eGFR was stratified into two groups: a main group with eGFR (60–80) mL/min/1.73 m^2^ and a control group with eGFR > 80 mL/min/1.73 m^2^. This stratification was defined for CKD-EPI 2021 following a series of comparative analyses between different eGFR categories using the Cochran–Mantel–Haenszel (CMH) test [20]. Statistical analysis and data visualization were performed using the Python programming language, version 3.10 (Python Software Foundation, Wilmington, DE, USA), executed in the Google Colaboratory environment (Google LLC, Mountain View, CA, USA). The following statistical methods were applied: the Shapiro–Wilk and D’Agostino-Pearson tests, Chi-square test and multivariable logistic regression. Data processing was performed using the pandas [21], scipy.stats [22], and statsmodels [23] libraries, and the plots were generated using the matplotlib [24] library. The level of statistical significance was set at *p* < 0.05. A Chi-square test initially evaluated the association between categorical variables. However, multivariable logistic regression served as the primary method to calculate Odds Ratios (ORs) and 95% Confidence Intervals (CIs) for each Lp(a) quartile relative to the reference group (Q1), after adjusting for major confounders (age, sex, diabetes, hypertension, and a complete lipid profile). The overall discriminatory performance of the model, built using the standardized CKD-EPI 2021 eGFR equation, was assessed via ROC/AUC analysis.

## 3. Results

### 3.1. Results of the Normality Tests for the Independent Variable Lp(a)

**Figure 1:**
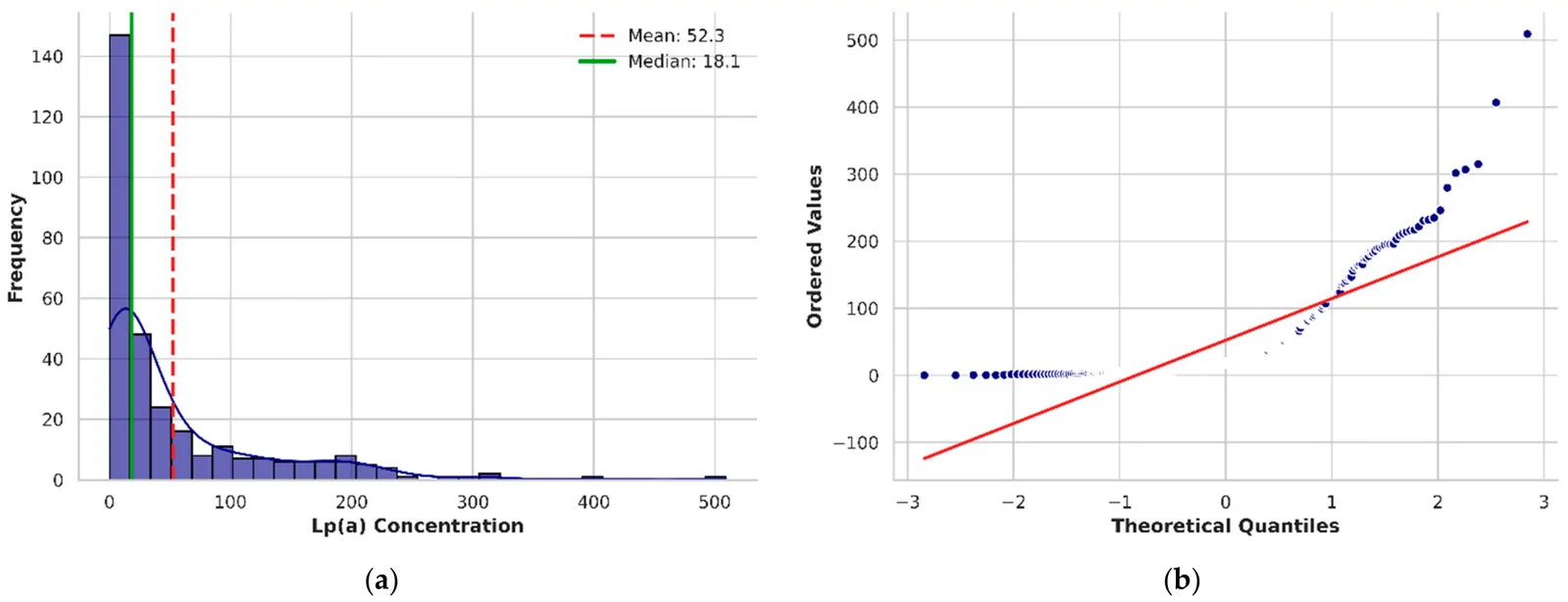
Assessment of the normality of distribution of serum Lp(a) concentrations. The histogram (**a**) illustrates the strongly right-skewed (asymmetric) distribution of Lp(a), where the red dashed line shows the mean and the green solid line represents the median. The Q-Q plot (**b**) further demonstrates the deviation from a normal distribution, as the ordered values (blue dots) diverge significantly from the theoretical normal line (red solid line).

The Shapiro–Wilk and D’Agostino-Pearson tests demonstrated a strong skewness in the Lp(a) distribution (Mean: 52.3 nmol/L, Median: 18.1 nmol/L), as well as the presence of outliers (Figure 1).

### 3.2. Definition of Target and Control Groups and Cohort Distribution According to eGFR Equations

To accurately assess the relationship between Lp(a) and early renal changes, we first defined the eGFR strata using the diagnostic standard, the CKD-EPI 2021 equation. Initially, the overall cohort was divided into three subgroups (I: 60–80 mL/min/1.73 m^2^; II: 80–90 mL/min/1.73 m^2^; III: >90 mL/min/1.73 m^2^), upon which the CMH method was applied to verify the relationship across Lp(a) quartiles, with Q1 set as the reference. The results of these comparisons are presented in Table 1.

The CMH test revealed no statistically significant difference between the second (eGFR 80–90 mL/min/1.73 m^2^) and third (eGFR > 90 mL/min/1.73 m^2^) groups (*p* = 0.927). Due to this lack of variance, they were combined into a single control group (eGFR > 80 mL/min/1.73 m^2^). The main target group thus consisted exclusively of patients with mildly reduced filtration (eGFR 60–80 mL/min/1.73 m^2^).

Based on this statistically validated stratification using CKD-EPI 2021, 172 patients (55.5%) were categorized into the main target group, and 138 patients (44.5%) formed the control group.

**Figure 2:**
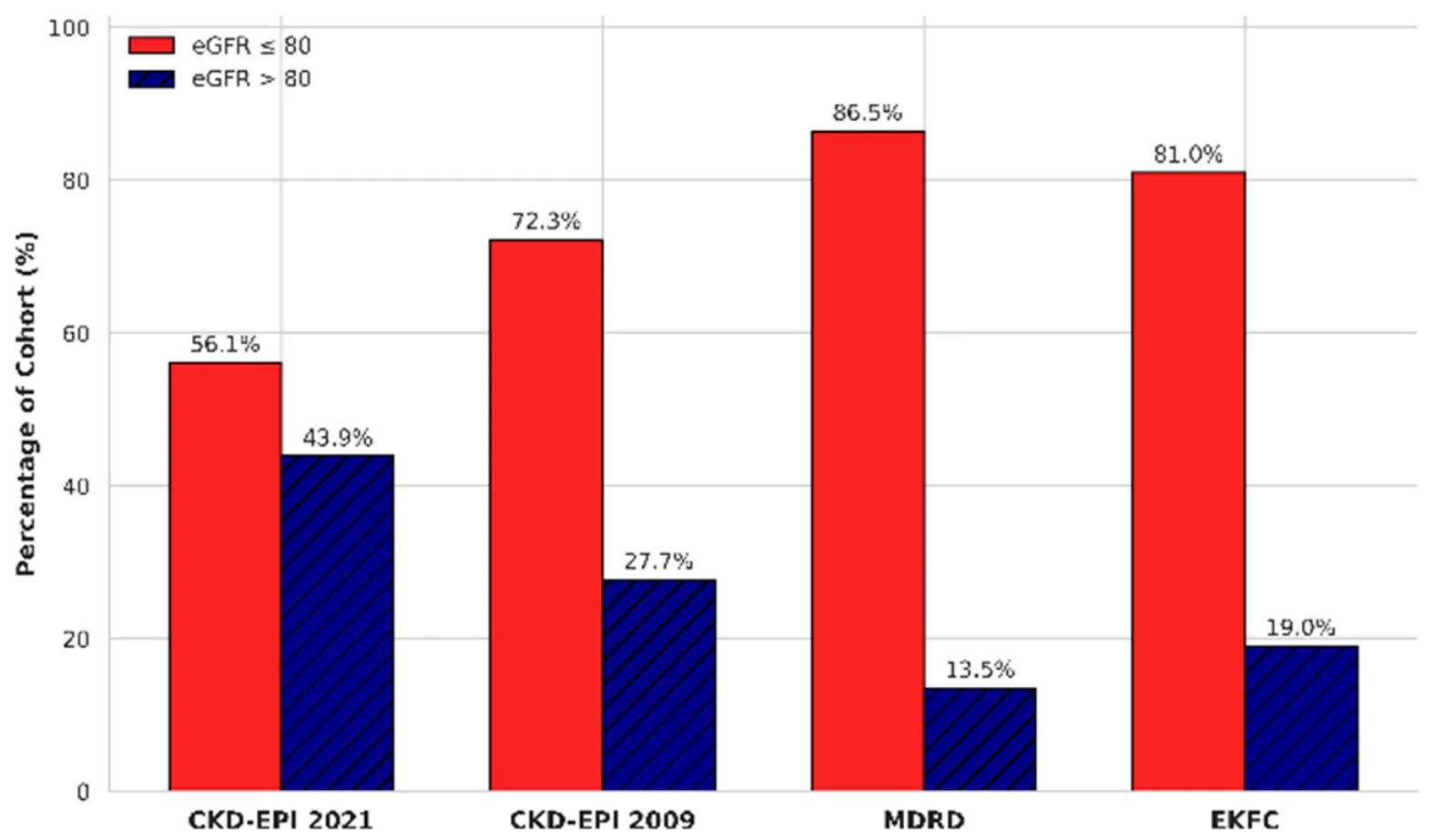
Percentage distribution of the cohort between the control (eGFR > 80 mL/min/1.73 m^2^) and the study (eGFR 60–80 mL/min/1.73 m^2^) group according to the equation used. Abbreviations: eGFR—estimated glomerular filtration rate; CKD-EPI—Chronic Kidney Disease Epidemiology Collaboration; MDRD—Modification of Diet in Renal Disease; EKFC—European Kidney Function Consortium.

After establishing the baseline groups, we evaluated how the application of alternative equations (CKD-EPI 2009, CKD-MDRD, and CKD-EKFC) redistributes the identical cohort (Figure 2).

When calculating the eGFR using these alternative equations, a substantial redistribution of our cohort occurred. This was most pronounced when using the CKD-MDRD equation: 86.5% of the cohort was categorized into the group with reduced eGFR (eGFR 60–80 mL/min/1.73 m^2^), which significantly impacted and depleted the control group.

### 3.3. Initial Non-Parametric Evaluation and Multivariable Logistic Regression Without Adjustment for Age and Sex

In the initial unadjusted analysis based on the CKD-EPI 2021 equation, the Chi-square test demonstrated a statistically significant association between Lp(a) quartiles and eGFR categories (Chi-square = 12.5887, *p* = 0.0056). Specifically, we observed a distinct variation in the distribution of patients with reduced filtration across the ascending Lp(a) groups. Cramer’s V of 0.20 indicates a weak to moderate strength of this association. This primary bivariate analysis confirms the presence of a relationship between Lp(a) concentrations and renal function. To further quantify the risk of reduced filtration and evaluate the independent predictive value of Lp(a), the data were subjected to multivariable logistic regression.

The results obtained following the application of multivariable logistic regression to the four eGFR models (defined by the type of equation used) are presented in Table 2.

**Figure 3:**
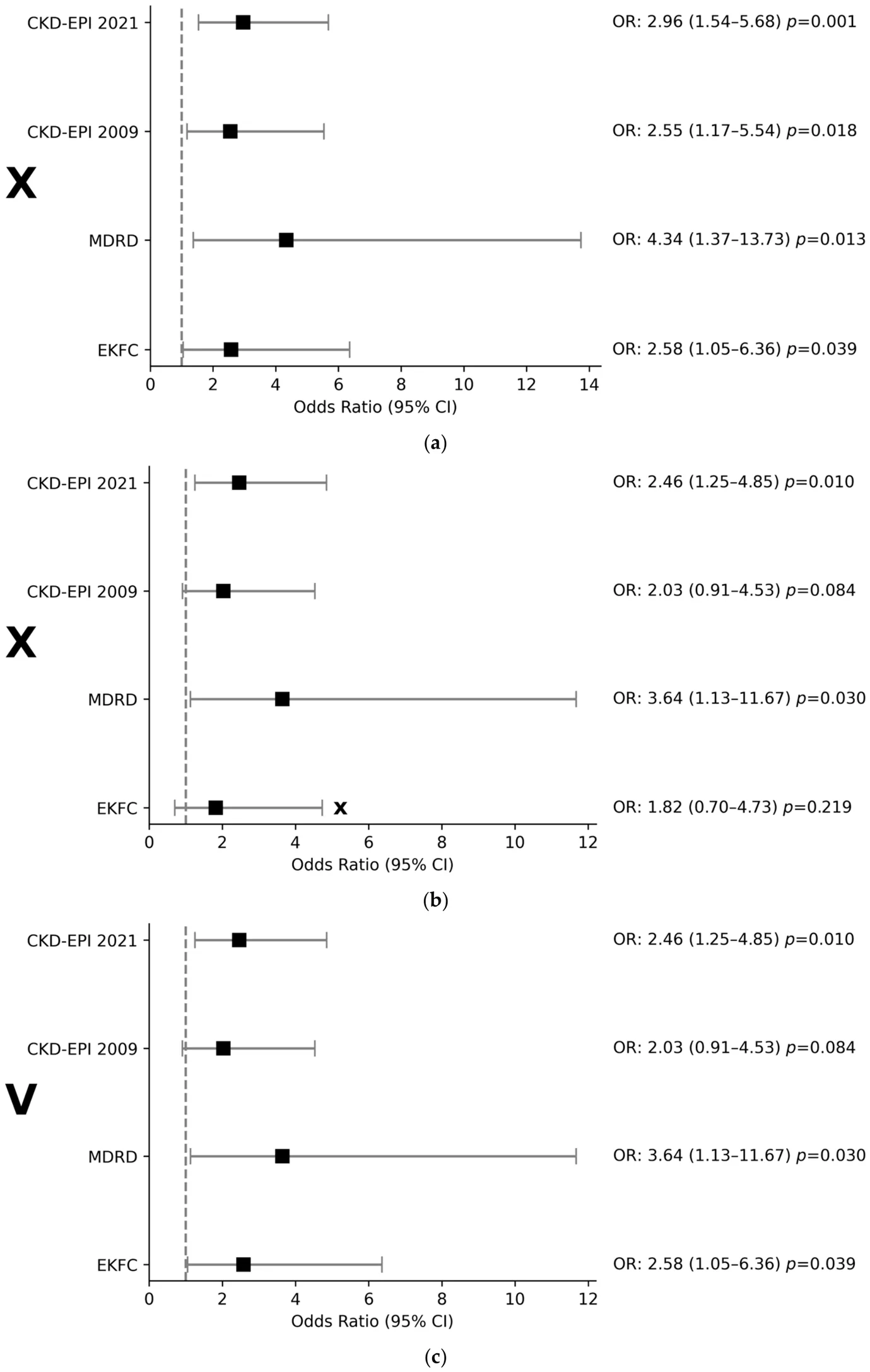
Forest Plots. omparative analysis of the association in Q4 across the assessment eGFR models. The graphs illustrate the odds ratios (OR) for patients in the highest quartile of Lp(a) to fall into the group with mildly reduced eGFR (eGFR 60–80 mL/min/1.73 m^2^). Panel (**a**)—Results after multivariate logistic regression without accounting for demographic confounding factors. Panel (**b**)—Results with age and sex included as a confounding factor. The “X” symbol denotes an incorrect statistical approach in both scenarios: Panel (**a**) lacks the mandatory demographic adjustments for CKD-EPI and MDRD, while Panel (**b**) incorrectly adjusts EKFC for age and sex, as these parameters are inherently embedded in the equation itself. Panel (**c**)—The correct comparative assessment: CKD-EPI 2021, CKD-EPI 2009, and MDRD are adjusted for age and sex, while EKFC remains unadjusted (indicated by “V”).

The analysis demonstrates a statistically significant association in the fourth quartile (Q4) across all eGFR groups (Figure 3a). The results from the application of CKD-EPI 2009 most closely approximate those obtained with CKD-EPI 2021, although the OR is lower. Regarding the EKFC equation, while the lower limit of the confidence interval borders on 1.0 (1.05), it still retains statistical significance. The result from the application of CKD-MDRD exhibits a markedly wide confidence interval (CI: 1.37–13.73), which indicates mathematical instability.

### 3.4. Cohort Age Distribution According to CKD-EPI 2021-Estimated eGFR

**Figure 4:**
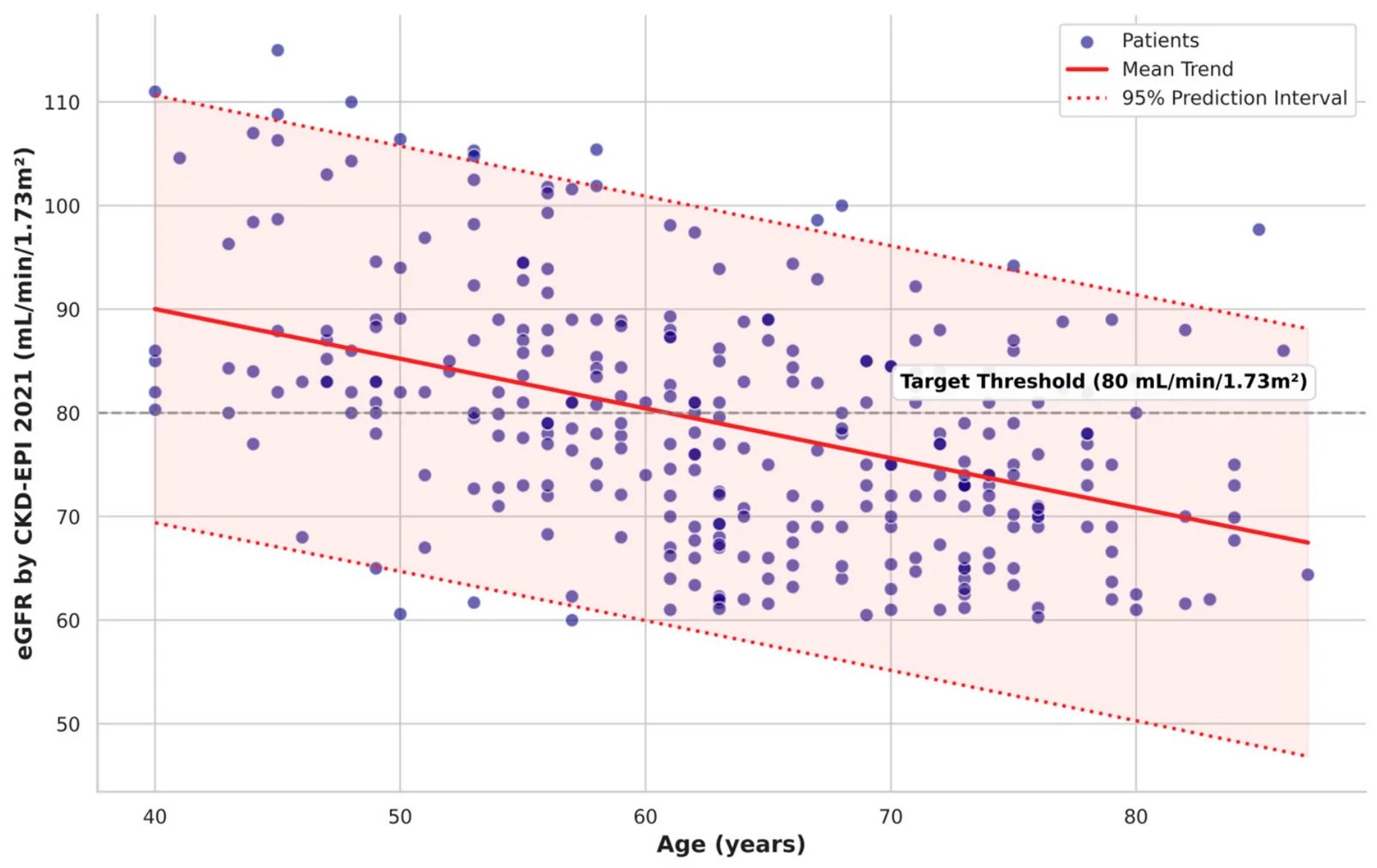
Relationship between age and eGFR calculated using the CKD-EPI 2021 equation. The scatter plot presents the distribution of individual eGFR values (blue dots) in patients of different ages. The red solid line reflects the mean linear regression trend, demonstrating the physiological decline of renal function with advancing age. The red dotted lines and the light red shaded area between them indicate the 95% prediction interval, illustrating the expected variation and the clinical range of eGFR values for the cohort. The horizontal grey dashed line marks the target threshold of 80 mL/min/1.73 m^2^, which is defined as the cutoff for the “gray zone” for our sample (60–80 mL/min/1.73 m^2^).

According to the recommendations of the National Kidney Foundation (NKF), the mean normal eGFR declines with age, i.e., there is a physiological dynamic. This is also observed in our cohort (Figure 4).

The observed pronounced association between age and eGFR provides the rationale for the statistical isolation of this effect.

### 3.5. Results of Multivariable Logistic Regression Analysis Adjusted for Age and Sex

To determine the independent effect of Lp(a) on eGFR, we included age and sex as confounding factors in the multivariable regression analysis and re-analyzed the four eGFR models (Table 3). Age was dichotomized at >63 years for the regression analysis as this represents the median age of our study cohort (age range 40–87).

Age demonstrated a statistically significant effect on the eGFR across all four groups. In contrast, sex (being male) did not show a statistically significant independent effect on eGFR in any of the models (*p* > 0.05). This lack of independent statistical significance is expected, as the physiological differences in muscle mass and creatinine generation between sexes are already mathematically integrated into the core architecture of all evaluated equations (e.g., via sex-specific coefficients in CKD-EPI and MDRD, or sex-specific Q-values in EKFC). For CKD-EKFC, a so-called over-adjustment is observed due to the age correction already incorporated into the equation [19] (OR = 13.79, 95% CI 5.28–36.04). The application of multivariable logistic regression to isolate the effect of age is inappropriate in this case (age is entered as a factor twice) (Figure 3b).

In the CKD-EPI 2021 category, the confounding effect of age and sex does not affect the statistical significance of the association in Q4 (*p* = 0.012), whereas in Q2 it loses its statistical significance (*p* = 0.094). The odds of reduced eGFR in Q4 remain more than two times higher (OR = 2.41) than those of the reference group. In the CKD-EPI 2009 group, we observe a loss of statistical significance in Q4 (*p* = 0.096). The effect of age on the association in Q4 of the CKD-MDRD group is negligible. It maintains its mathematical instability (95% CI 1.08–11.27) (Figure 3b).

### 3.6. Assessment of Additional Clinical Confounding Factors

To isolate the independent effect of Lp(a) on eGFR in the main group (eGFR 60–80 mL/min/1.73 m^2^) and to evaluate the influence of confounding factors, four progressive multivariable logistic regression models were constructed (Table 4). The threshold values used to dichotomize the standard lipid profile variables (Total Cholesterol < 5.0 mmol/L, LDL < 3.0 mmol/L, HDL > 1.0 mmol/L for males and >1.2 mmol/L for females, and Triglycerides < 1.7 mmol/L) were adopted in accordance with the 2025 European Heart Journal guidelines [25]. Other confounding factors were entered into the model as dichotomous variables: age (>63 years), male sex, and the presence of hypertension or diabetes.

Utilizing the CKD-EPI 2021 equation, our fully adjusted multivariable logistic regression analysis demonstrated that only the highest quartile of Lp(a) (Q4) is significantly associated with mildly reduced eGFR, independent of confounding factors. Conversely, across the other evaluated eGFR models, this association proved to be either statistically non-significant or mathematically unstable.

**Figure 5:**
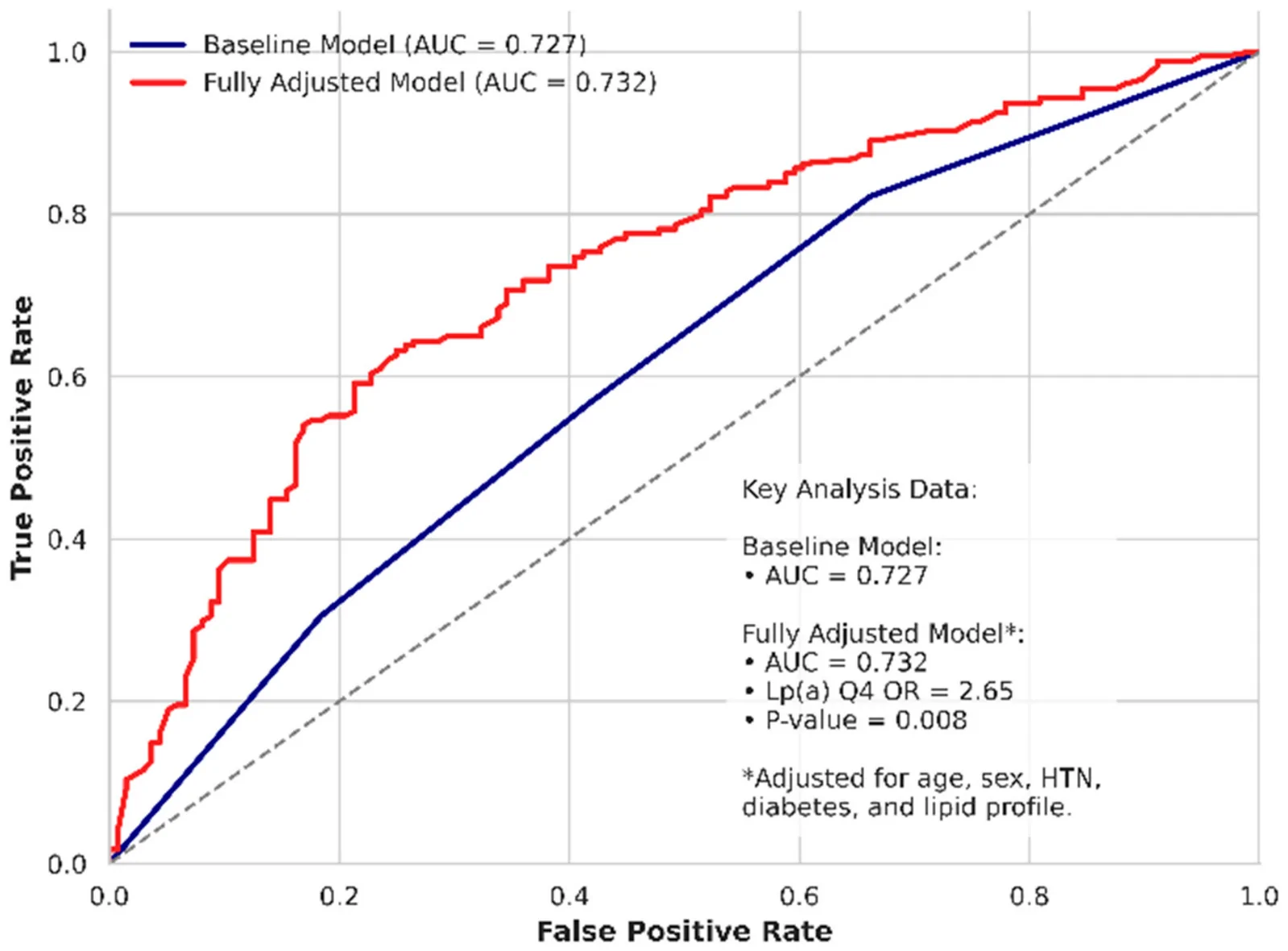
Receiver Operating Characteristic (ROC) curves comparing the discriminatory ability of baseline and fully adjusted logistic regression models (using the CKD-EPI 2021 framework). The baseline model (blue line) evaluates the direct predictive capacity of Lp(a) quartiles for mildly decreased eGFR. The fully adjusted model (red line) is corrected for age, sex, hypertension, diabetes, and the full lipid profile. This full adjustment yielded an improved Area Under the Curve (AUC) of 0.732, with the highest Lp(a) quartile (Q4) remaining an independent, statistically significant predictor. The grey dashed line represents the reference line (AUC = 0.5). The asterisk (*) denotes the variables included in the fully adjusted model.

To evaluate the diagnostic performance and discriminatory power of the assessment framework, we calculated the Area Under the Receiver Operating Characteristic Curve (AUC) for the CKD-EPI 2021 equation. The baseline model (without confounders included) achieved an AUC of 0.727, indicating acceptable discriminative ability. Following the comprehensive integration of all clinical confounders—including age, sex, hypertension, diabetes, and the complete lipid profile—the fully adjusted model demonstrated an improved and robust predictive performance, yielding an AUC of 0.732 (Figure 5). These findings confirm the diagnostic utility and stability of the framework when simultaneously accounting for traditional metabolic and vascular risk factors.

Analysis of the individual confounders revealed that metabolic parameters, including diabetes and the complete lipid profile (total cholesterol, LDL, HDL, and triglycerides), failed to reach independent statistical significance in any of the models (all *p* > 0.05). In contrast, hypertension maintained its status as a robust, independent predictor of mildly reduced renal filtration.

## 4. Discussion

The present comparative analysis confirms that the choice of equation for eGFR is a critical methodological factor when constructing assessment models for biomarkers. Within the investigated “gray zone” of low–normal filtration (60–80 mL/min/1.73 m^2^), the choice of mathematical formula directly determines the structure of the dependent variable. Neglecting this methodological detail may lead to a distortion of regression models and obscure the actual pathological effect of factors such as serum Lp(a).

The increased risk of mildly reduced filtration demonstrated in the CKD-EPI 2021 model for individuals in the highest quartile (Q4 > 40.45 nmol/L) aligns with current epidemiological data. This observed dependence reflects a complex pathophysiological relationship. The underlying pathophysiology is inherently bidirectional, as renal dysfunction impairs the catabolism of Lp(a), causing its systemic elevation [7,8], while high lipid levels reciprocally exert deleterious effects on the glomeruli via pro-inflammatory and pro-thrombotic mechanisms [9,10].

Prospective and cross-sectional studies in independent cohorts also confirm that a mild decline in eGFR values within the 60–89 mL/min/1.73 m^2^ range is directly linked to elevated baseline serum Lp(a) levels [26,27,28,29]. This underscores the clinical necessity of identifying these borderline conditions in practice before CKD [30,31].

The primary cause of the statistical discrepancies among the four tested unadjusted models is rooted in the systematic bias of the older equations (CKD-MDRD, CKD-EPI 2009) and the CKD-EKFC equation. Due to their tendency to underestimate actual eGFR in healthy individuals or early-stage patients, these equations induce an artificial redistribution of the cohort [32]. A substantial proportion of participants are misclassified from the control group into the target group with reduced eGFR. This artificial expansion of the sample dilutes the specific effect of Lp(a) and reduces the statistical power of the regression analysis.

Introducing age and sex as a confounder reveals fundamental differences in the mathematical architecture of the equations. Only the CKD-EPI 2021 standard effectively distinguishes the physiological aging of the renal parenchyma and sex-related differences from the pathological influence of Lp(a), maintaining the stability and significance of the model (*p* = 0.012). Conversely, applying this simultaneous adjustment for age and sex to the CKD-EKFC model leads to the phenomenon of overadjustment. The fundamental reason is that EKFC is a Full Age Spectrum (FAS) equation, and it already incorporates a built-in parameter for the age-scaling of serum creatinine alongside a sex-specific normalization constant (the Q-factor, set at 0.70 for females and 0.90 for males). This design inherently accounts for both the non-linear physiological decline in filtration after the age of 40 and the demographic variations in creatinine generation [19]. Consequently, re-introducing age and sex as independent variables in the multivariable logistic regression generates severe multicollinearity. The model becomes mathematically unstable, artificially inflates the OR for age (OR = 13.79), and completely obliterates the true independent effect of the highest Lp(a) quartile (Q4). Our multivariable analyses demonstrate that while hypertension is a significant independent risk factor, it does not eliminate the pathogenic significance of high Lp(a) levels. Specifically, when utilizing the standardized CKD-EPI 2021 equation, the association between the highest Lp(a) quartile (Q4) and eGFR remains independent of concomitant metabolic or vascular disorders, such as diabetes, hypertension, and standard lipid profile variations.

Study Limitations: A potential limitation of this study is the use of consecutive sampling, which is a non-probability sampling technique and may introduce a degree of selection or temporal bias. However, this approach is widely accepted and highly practical in clinical research. To minimize these biases and ensure the cohort’s representativeness, strict adherence to the pre-defined inclusion and exclusion criteria was maintained throughout the enrollment period. Additionally, the relatively modest sample size and the absence of external validation necessitate cautious interpretation of the findings. Certain potential confounding factors, such as body mass index (BMI) were not included in the final regression models due to data constraints, which represents another limitation of our assessment.

## 5. Conclusions

The choice of eGFR equation is of fundamental importance in constructing assessment models aimed at determining the independent effect of biomarkers on renal function, and in comparing results across different scientific studies. The underestimation of eGFR by the older equations and by the European consortium’s CKD-EKFC [32] may obscure the true pathological association of the investigated parameters. In our case, the conclusions are:

- CKD-EPI 2009 does not fully isolate age as a confounding factor. This limitation must be taken into consideration in comparative analyses and meta-analyses that include studies conducted before the new CKD-EPI 2021 equation was established.
- The mathematical instability of CKD-MDRD in our case is due to the “depletion” of the control group resulting from the artificial redistribution of participants. We did not consider the lack of sufficient data for a robust statistical analysis to be a major limitation, as this equation is rarely used in routine clinical practice.
- EKFC is in the process of validation. When constructing assessment models seeking new biomarkers causing renal injury, it must be used without applying statistical adjustment for age and sex (as these demographic parameters are inherently embedded in the equation itself). This must also be taken into consideration when making comparisons with studies in which CKD-EPI 2021 is used for the assessment of renal function (where accounting for age as a confounding factor is mandatory) (Figure 3c).

Our comparative analysis demonstrated that the new CKD-EPI 2021 equation provided the most stable and reliable results in patients with a low-normal eGFR within our cohort. It effectively isolates the independent effect of Lp(a) Q4 from age, sex, and a large majority of the established clinical confounders related to kidney function. Its utilization, together with the laboratory analysis methods recommended by the IFCC, enables the identification of high-risk groups, such as the Lp(a) Q4 group. Distinguishing the physiological changes in aging from true renal pathology is fundamental for validating assessment models and accurately stratifying the risk of new biomarkers. Therefore, strict adherence to the standardized CKD-EPI 2021 equation is essential for the reliability of future meta-analyses and clinical guidelines, ultimately facilitating the early detection and timely clinical management of asymptomatic patients.

## Figures and Tables

**Table 1:** Results of the CMH and Woolf tests verifying the relationship between Lp(a) quartiles across different eGFR group comparisons.

eGFR Comparison Groups	Lp(a) Quartiles (ORs vs. Q1)			CMH Test		Woolf Test
(mL/min/1.73 m^2^)	Q2	Q3	Q4	Overall OR (95% CI)	*p*-Value	*p*-Value
80–90 vs. >90	0.963	0.673	1.750	0.984 (0.553–1.752)	0.927	0.456
60–80 vs. >90	2.077	1.529	4.787	2.301 (1.333–3.971)	0.003	0.282
60–80 vs. 80–90	2.159	2.271	2.735	2.376 (1.572–3.589)	<0.001	0.889

Abbreviations: CMH—Cochran–Mantel–Haenszel; eGFR—estimated glomerular filtration rate; Lp(a)—lipoprotein(a); OR—odds ratio; Q—quartile. Notes: The Cochran–Mantel–Haenszel (CMH) test was applied to evaluate the overall association. The Woolf test was used to assess the homogeneity of odds ratios across the defined strata. Statistical significance was set at *p* < 0.05.

**Table 2:** Results of the association between Lp(a) and eGFR, calculated using the four equations, obtained after applying multivariable logistic regression.

	CKD-EPI 2021		CKD-EPI 2009		CKD-MDRD		CKD-EKFC	
	OR 95% CI	*p*-Value	OR 95% CI	*p*-Value	OR 95% CI	*p*-Value	OR 95% CI	*p*-Value
Q1	REF	-	REF	-	REF	-	REF	-
Q2	1.92 (1.01–3.63)	0.046	1.14 (0.57–2.26)	0.715	1.74 (0.71–4.27)	0.223	1.31 (0.6–2.86)	0.503
Q3	1.86 (0.98–3.51)	0.056	0.86 (0.44–1.67)	0.651	1.05 (0.47–2.36)	0.897	0.90 (0.43–1.88)	0.781
Q4	2.96 (1.54–5.68)	<0.001	2.55 (1.17–5.54)	0.018	4.34 (1.37–13.73)	0.013	2.58 (1.05–6.36)	0.039

Abbreviations: OR—odds ratio; CI—confidence interval; REF—reference category; Lp(a)—lipoprotein(a); eGFR—estimated glomerular filtration rate; CKD-EPI—Chronic Kidney Disease Epidemiology Collaboration; MDRD—Modification of Diet in Renal Disease; EKFC—European Kidney Function Consortium; Q—quartile.

**Table 3:** Results of the assessment of the independent effect of Lp(a) on eGFR, calculated using the four equations and after adjusting for age and sex as confounding factors.

Quartiles	CKD-EPI 2021		CKD-EPI 2009		CKD-MDRD		CKD-EKFC	
	OR 95% CI	*p*-Value	OR 95% CI	*p*-Value	OR 95% CI	*p*-Value	OR 95% CI	*p*-Value
Q1	REF	-	REF	-	REF	-	REF	-
Age (>63)	3.21 (1.98–5.21)	<0.001	3.77 (2.13–6.67)	<0.001	2.62 (1.25–5.51)	0.010	13.79 (5.28–36.04)	0.001
Sex (men)	0.67 (0.41–1.08)	0.100	0.73 (0.43–1.24)	0.246	0.53 (0.27–1.05)	0.068	0.67 (0.36–1.26)	0.215
Q2	1.77 (0.91–3.45)	0.094	0.99 (0.49–2.04)	0.988	1.52 (0.61–3.80)	0.368	1.08 (0.46–2.51)	0.858
Q3	1.54 (0.79–3.00)	0.206	0.65 (0.32–1.33)	0.242	0.82 (0.35–1.91)	0.649	0.58 (0.25–1.33)	0.193
Q4	2.41 (1.22–4.78)	0.012	1.98 (0.88–4.45)	0.096	3.49 (1.08–11.27)	0.037	1.76 (0.67–4.59)	0.249

Abbreviations: OR—odds ratio; CI—confidence interval; REF—reference category; Lp(a)—lipoprotein(a); eGFR—estimated glomerular filtration rate; CKD-EPI—Chronic Kidney Disease Epidemiology Collaboration; MDRD—Modification of Diet in Renal Disease; EKFC—European Kidney Function Consortium; Q—quartile.

**Table 4:** Results of the fully adjusted multivariable logistic regression analysis evaluating the independent association between Lp(a) quartiles and mildly reduced eGFR across the four assessment equations.

Quartiles	CKD-EPI 2021		CKD-EPI 2009		CKD-MDRD		CKD-EKFC	
	OR 95% CI	*p*-Value	OR 95% CI	*p*-Value	OR 95% CI	*p*-Value	OR 95% CI	*p*-Value
Q1	REF	-	REF	-	REF	-	REF	-
Age	3.21 (1.98–5.21)	<0.001	3.77 (2.13–6.67)	<0.001	2.62 (1.25–5.51)	0.010	13.79 (5.28–36.04)	0.001
Sex	0.67 (0.41–1.08)	0.100	0.73 (0.43–1.24)	0.246	0.53 (0.27–1.05)	0.068	0.67 (0.36–1.26)	0.215
Hypertension	2.43 (1.34–4.08)	0.003	2.53 (1.38–4.64)	0.003	2.08 (0.95–4.57)	0.068	3.93 (1.85–8.35)	<0.001
Diabetes	0.87 (0.49–1.55)	0.640	0.58 (0.30–1.11)	0.098	0.51 (0.23–1.15)	0.104	0.41 (0.18–0.93)	0.033
TC	1.18 (0.59–2.37)	0.640	1.40 (0.65–3.0)	0.389	1.68 (0.62–4.51)	0.306	1.70 (0.68–4.23)	0.254
LDL	0.90 (0.404–1.84)	0.777	1.10 (0.50–2.42)	0.819	0.92 (0.33–2.56)	0.880	1.01 (0.39–2.63)	0.984
HDL	1.10 (0.58–2.06)	0.776	1.11 (0.54–2.25)	0.779	0.84 (0.32–2.22)	0.720	0.76 (0.31–1.85)	0.550
TG	0.72 (0.41–1.28)	0.265	0.51 (0.26–0.98)	0.043	0.32 (0.13–0.83)	0.018	0.52 (0.23–1.16)	0.111
Q2	1.97 (0.98- 3.97)	0.057	1.10 (0.52–2.34)	0.806	1.86 (0.71–4.86)	0.207	1.16 (0.47–2.87)	0.750
Q3	1.72 (0.85- 3.45)	0.129	0.73 (0.35–1.53)	0.402	0.97 (0.41–2.34)	0.954	0.61 (0.25–1.48)	0.275
Q4	2.65 (1.29–5.43)	0.008	2.14 (0.92–4.97)	0.078	3.79 (1.13–12.69)	0.031	1.74 (0.63–4.83)	0.289

Abbreviations: CI—confidence interval; CKD-EPI—Chronic Kidney Disease Epidemiology Collaboration; EKFC—European Kidney Function Consortium; HDL—high-density lipoprotein; LDL—low-density lipoprotein; MDRD—Modification of Diet in Renal Disease; OR—odds ratio; Q—quartile; REF—reference category; TC—total cholesterol; TG—triglycerides.

## Data Availability

The data presented in this study are available on request from the corresponding author. The data are not publicly available due to institutional privacy and ethical restrictions regarding patient confidentiality.

## Abbreviations

The following abbreviations are used in this manuscript:

AUC	Area Under the Curve
BMI	Body Mass Index
CI	Confidence Interval
CKD	Chronic kidney disease
CKD-EPI	Chronic Kidney Disease Epidemiology Collaboration
CMH	Cochran–Mantel–Haenszel
eGFR	estimated Glomerular Filtration Rate
EKFC	European Kidney Function Consortium
FAS	Full Age Spectrum
HDL	High-Density Lipoprotein
IDMS	Isotope Dilution Mass Spectrometry
IFCC	International Federation of Clinical Chemistry and Laboratory Medicine
LDL	Low-Density Lipoprotein
Lp(a)	Lipoprotein(a)
MDRD	Modification of Diet in Renal Disease
OR	Odds Ratio
Q	Quartile
REF	Reference category
ROC	Receiver Operating Characteristic
TC	Total Cholesterol
TG	Triglycerides
